# Exfoliative Erythroderma Revealing Primary Central Nervous System Diffuse Large B-Cell Lymphoma

**DOI:** 10.7759/cureus.106892

**Published:** 2026-04-12

**Authors:** Iman Bouchelkia, Theodore Rosen

**Affiliations:** 1 Biomedical Sciences, Tilman J. Fertitta Family College of Medicine, Houston, USA; 2 Dermatology, Michael E. DeBakey Veterans Affairs Medical Center, Baylor College of Medicine, Houston, USA

**Keywords:** exfoliative dermatitis, exfoliative erythroderma, occult malignancy, primary central nervous system lymphoma (pcnsl), refractive disease

## Abstract

Exfoliative erythroderma is a severe inflammatory skin condition that may result from pre-existing dermatoses, medications, or underlying malignancy. Although malignancy-associated cases are uncommon, persistent and treatment-resistant erythroderma should raise concern for an occult cancer. We report the case of an 82-year-old man with a one-year history of diffuse pruritic erythema and scaling involving nearly the entire body surface area that remained refractory to multiple dermatologic and immunosuppressive therapies. Initial evaluation for cutaneous lymphoma and systemic malignancy was negative. Months later, the patient developed a generalized seizure, and a brain MRI revealed a right frontal lobe mass. Biopsy later confirmed Epstein-Barr virus-positive primary central nervous system diffuse large B-cell lymphoma. This case highlights persistent exfoliative erythroderma as a potential paraneoplastic manifestation of an underlying malignancy and emphasizes the importance of continued evaluation for occult cancer when erythroderma remains unexplained and refractory to treatment.

## Introduction

Exfoliative erythroderma (EE) is a skin condition where greater than 90% of the body is scaling, red, and inflamed [1]. There are many potential causes of EE, including idiopathic causes, exacerbation of pre-existing dermatosis, drug reaction, and even malignancy [2]. The disruption of the skin barrier in EE leads to increased transepidermal water loss, impaired thermoregulation, and increased patients’ susceptibility to infections. This can result in a life-threatening condition, especially in older patients. 

Although the exact pathogenesis of EE is unknown, it has been speculated that the condition is secondary to a complex interaction of cytokines and cellular adhesion molecules [3]. At the cellular level, widespread immune activation leads to accelerated epidermal turnover, resulting in diffuse scaling and barrier dysfunction. The clinical presentation of EE is often nonspecific and does not clearly point to the underlying cause, so recognizing and treating the primary disease is crucial to improving patient outcomes. 

While most cases of EE are attributed to inflammatory dermatoses or medications, a smaller but clinically significant proportion are associated with underlying malignancy. Nearly 1% of patients diagnosed with EE have an underlying malignancy [1]. Particular cancers such as cutaneous T cell lymphoma, B cell chronic lymphocytic leukemia, lung cancer, and gastrointestinal malignancies are strongly associated with EE [2,4,5]. In certain cases, the cutaneous symptoms may predate the diagnosis of malignancy and may be resistant to traditional dermatologic therapy, making identification of the underlying cancer challenging. Although hematologic malignancies are more commonly reported in association with EE, involvement related to primary central nervous system tumors is exceedingly rare. Our case presents a patient with persistent, treatment-resistant EE that ultimately led to the diagnosis of primary central nervous system diffuse large B-cell lymphoma. According to the available literature, this presentation is rarely described and represents a unique manifestation of primary central nervous system lymphoma.

## Case presentation

An 82-year-old man with a medical history significant for hypertension, chronic kidney disease, dyslipidemia, deep venous thrombosis on chronic enoxaparin therapy, and benign prostatic hypertrophy presented with a one-year history of progressive pruritus and diffuse erythema with scaling involving nearly the entire body surface area. His medications, including atorvastatin, carvedilol, enoxaparin, tamsulosin, and over-the-counter melatonin, had remained the same for several years. He denied any new prescription medications, supplements, or topical exposures before the onset of his symptoms.

**Figure 1:**
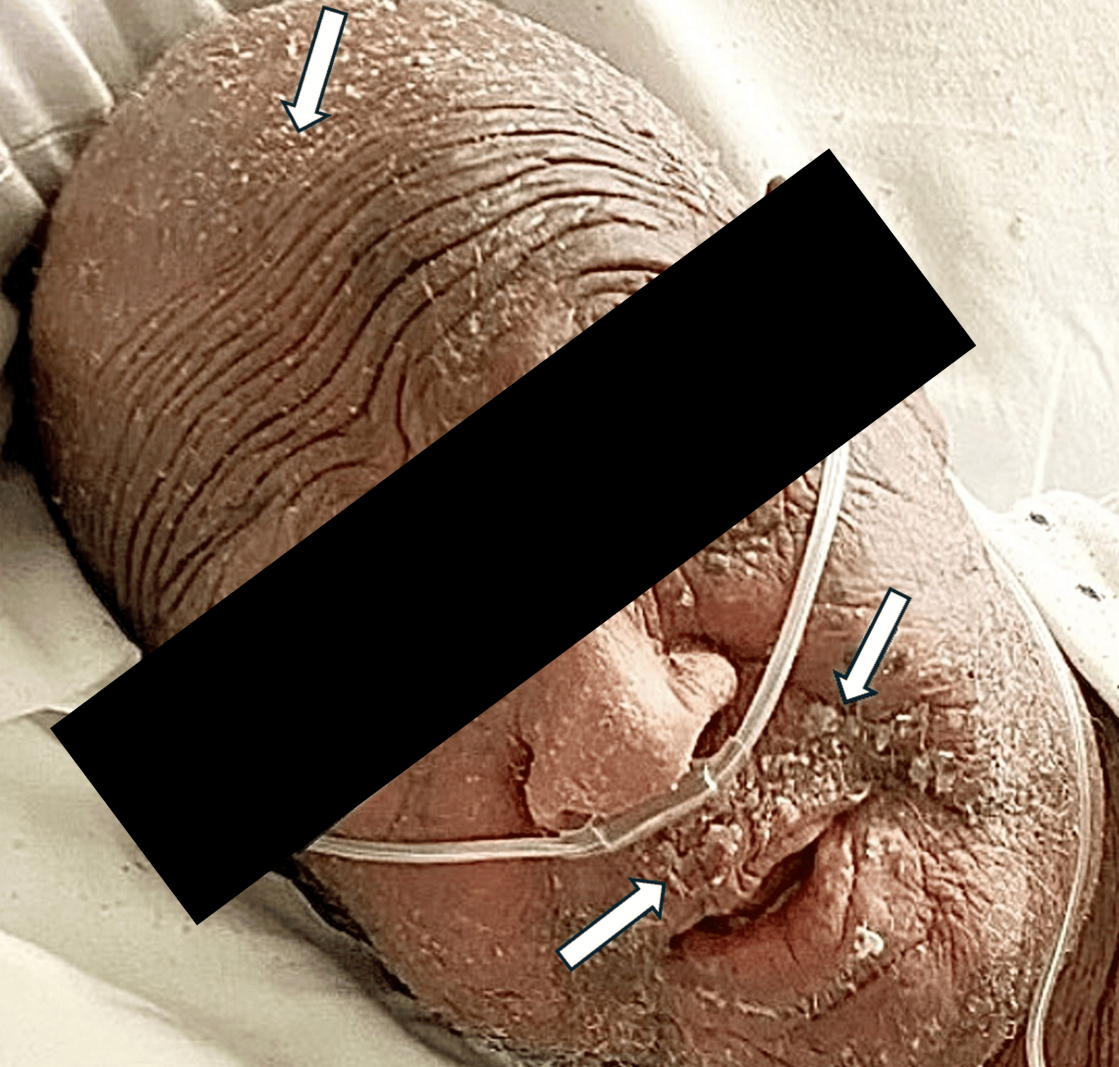
Facial involvement of exfoliative erythroderma demonstrating diffuse erythema with scaling

**Figure 2:**
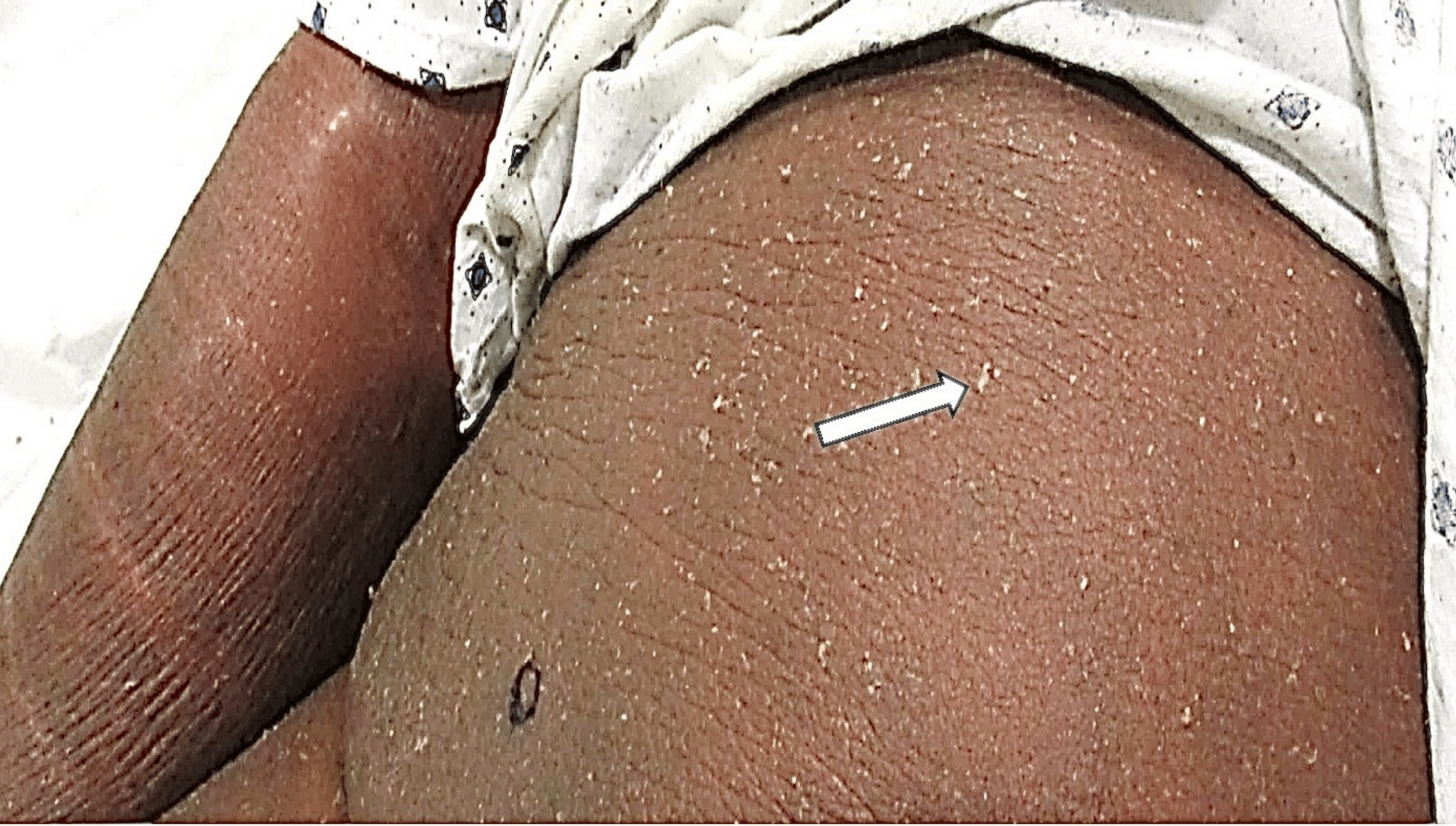
Diffuse erythema with prominent scaling involving the abdomen and upper extremities

On examination, he had generalized erythema and scaling consistent with EE (Figures 1, 2). Initial laboratory evaluation showed chronic renal impairment with a creatinine of 2.2 and a blood urea nitrogen of 33. His complete blood count was notable for eosinophilia of 10.8%. Liver function tests were normal. Given the extent and severity of his skin findings, multiple punch biopsies were performed over time. All four demonstrated spongiotic dermatitis with eosinophils, consistent with an eczematous process. He was treated with topical corticosteroids and moisturization without improvement. As his symptoms continued, systemic medication was started, including dupilumab, tralokinumab, methotrexate, systemic corticosteroids, and mycophenolate mofetil, and narrow band ultraviolet B phototherapy. Despite these therapies, his erythroderma remained diffuse and symptomatic.

Because of the refractory character of his condition, additional investigation was conducted to exclude cutaneous T cell lymphoma and systemic malignancy. T cell receptor gene rearrangement investigations were negative, and the peripheral smear did not identify Sézary cells. ​​Upper and lower endoscopy were unremarkable. No tumors or lymphadenopathy were visible on chest, abdominal, or pelvic computed tomography images. No underlying cause was found. Over the following months, his eosinophil count increased, eventually rising to 32.8%.

**Figure 3:**
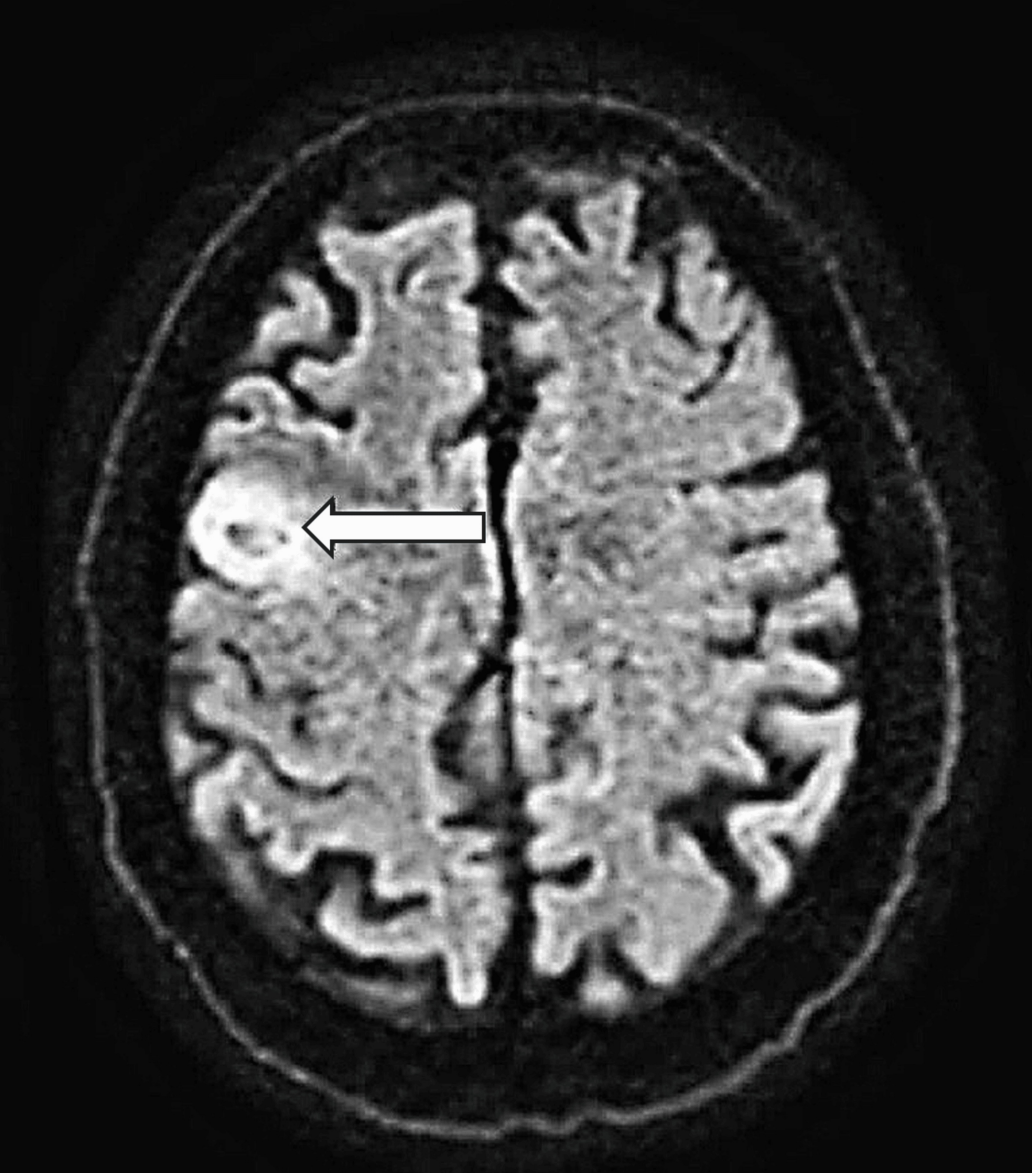
Brain MRI demonstrating a right frontal lobe lesion

**Figure 4:**
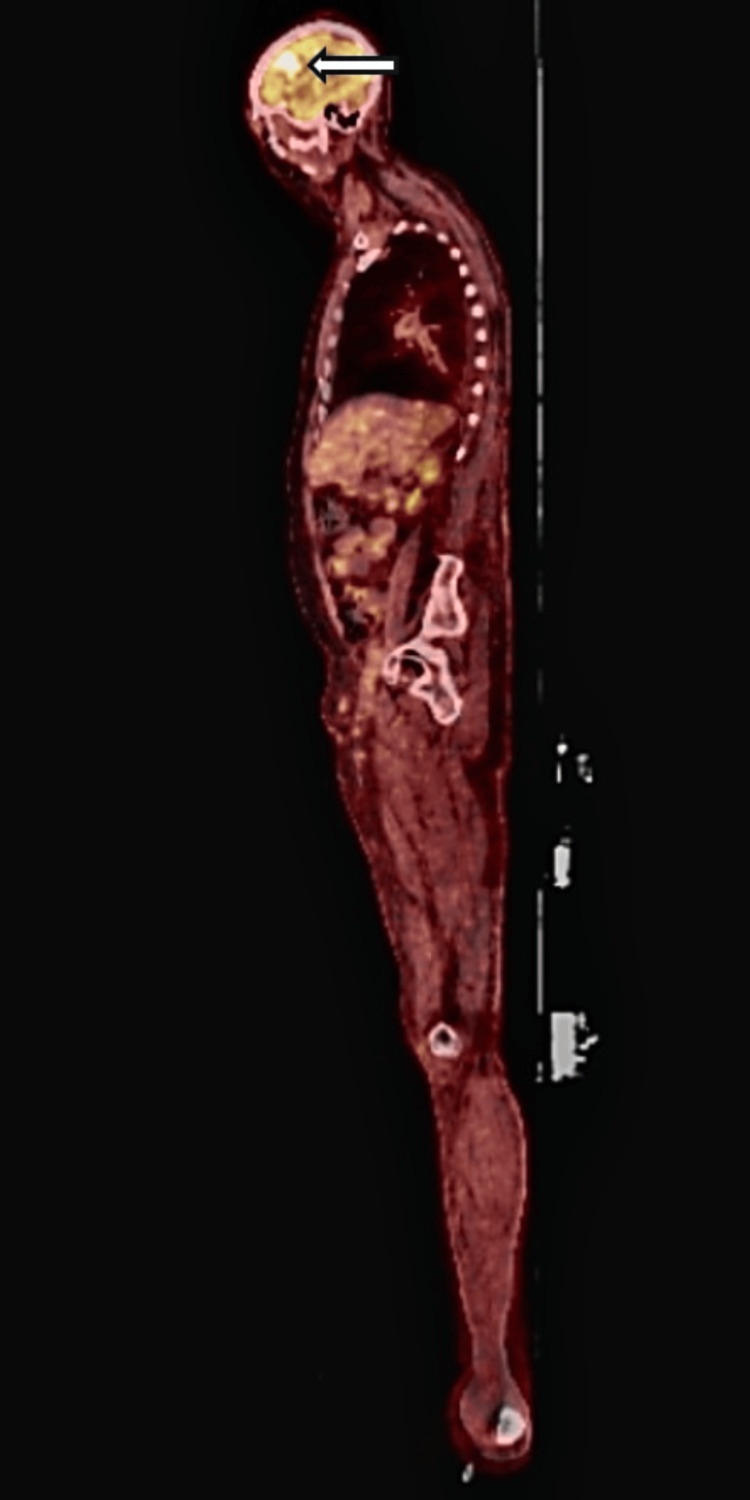
PET/CT showing an FDG-avid lesion in the right frontal cortex approximately 60 minutes after intravenous administration of 10.56 mCi of FDG. No metastasis was present. FDG: F18-fluorodeoxyglucose

**Figure 5:**
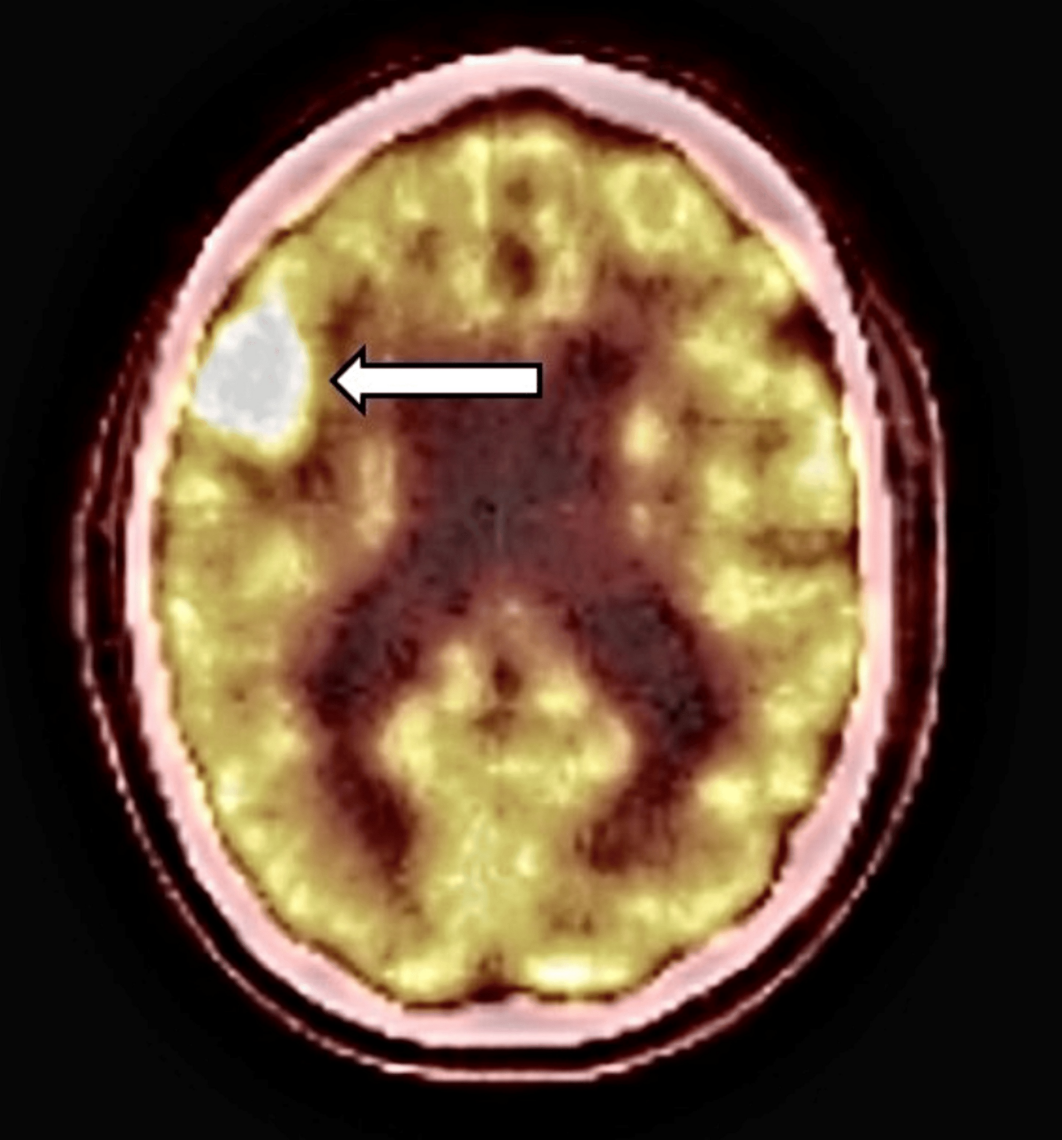
PET/CT view demonstrating increased F18-fluorodeoxyglucose (FDG) uptake in the right frontal cortical lesion.

Several months after his skin disease failed to respond to treatment, he experienced his first generalized seizure. Magnetic resonance imaging (MRI) of the brain revealed a right frontal lobe mass measuring 1.9 x 2.6 cm (Figure 3). Positron emission tomography (PET) demonstrated fluorodeoxyglucose avidity within the lesion in the right frontal cortex (Figures 4, 5). Repeat imaging one month later showed rapid interval growth to 3.2 x 3.4 cm. A stereotactic needle biopsy was done and revealed Epstein-Barr virus-positive primary central nervous system diffuse large B-cell lymphoma. Immunohistochemical analysis showed positivity for CD20, PAX 5, and BCL 2, rare expression of CD30 and CD23, and negativity for CD3, CD5, CD10, cyclin D1, BCL 6, c Myc, TdT, and CD34. The Ki-67 proliferation index was approximately 70%, consistent with a highly proliferative tumor. 

The patient underwent treatment with methotrexate and rituximab-based therapy, followed by whole brain radiotherapy and later ibrutinib with nivolumab. Despite aggressive treatment, the brain lesion progressed. His EE persisted throughout his oncologic course, with only temporary relief of pruritus from frequent emollient use, and almost two years after the initial presentation, he died following aspiration pneumonia.

## Discussion

Primary central nervous system lymphoma is an aggressive extranodal non-Hodgkin lymphoma. It is a rare illness that accounts for around 4% of newly diagnosed CNS malignancies, with an annual incidence rate of 0.5 per 100,000 in the United States [6]. The disease is primarily found in the brain, leptomeninges, and spinal cord, and can present with neurological changes. Because the disease is typically restricted to the central nervous system, patients may not show common systemic signs of lymphoma, which can make early diagnosis more difficult. In rare circumstances, however, malignancies may initially present with dermatologic manifestations.

Erythroderma is defined by persistent, widespread redness of the skin involving more than 90 percent of the body surface area, typically accompanied by variable degrees of scaling [7]. Its intensity and wide differential can pose serious diagnostic and treatment issues. The majority of instances are linked to exacerbations of preexisting dermatoses, drug responses, and malignancy [8]. In malignancy-associated EE, the dermatological findings may appear before the underlying cancer is found and are frequently resistant to conventional therapy. Hematologic malignancies, particularly cutaneous T cell lymphoma, are one of the most frequent cancers linked to EE [8]. In some patients, erythroderma may develop before the underlying malignancy is diagnosed and can represent a paraneoplastic skin manifestation [9]. Similar presentations have been described in other malignancies, including a reported case of prostate adenocarcinoma in which exfoliative dermatitis occurred as a paraneoplastic syndrome [10]. The cutaneous abnormalities are likely to emerge from cytokine-mediated or immune-dysregulated pathways caused by the underlying malignancy [9]. 

The majority of malignancy-related cases are linked to cancers that present with systemic symptoms. However, tumors that are limited to a certain anatomic region may go undetected during initial diagnostic evaluation. Primary central nervous system lymphoma is an important example of this pattern because the disease is frequently limited to intracranial structures and does not produce peripheral lymphadenopathy or abnormal hematologic findings that would otherwise raise suspicion during routine malignancy screening [6]. This diagnostic issue was represented in our case, where the underlying cancer was not detected during the first workup and only became apparent when the patient developed neurologic symptoms that required focused neuroimaging. This phenomenon has been described in erythroderma literature, where the underlying cause may go undetected and only becomes apparent with disease progression. To our knowledge, this is the only instance of EE associated with primary central nervous system lymphoma, emphasizing the necessity of evaluating occult malignancy when erythroderma continues without a known cause.

An additional feature in this case was the refractory nature of the EE despite standard dermatologic management. When EE persists after adequate treatment, the possibility of an underlying malignancy should be explored, even if the first assessment is negative. Histopathology is a valuable technique for diagnosing the etiology of erythroderma and should be performed and repeated at different sites and stages of the disease. In fact, it is an important diagnostic tool in erythroderma, contributing to the final diagnosis in approximately 55-66% of cases [11]. For this reason, early biopsy and various sampling from different sites or stages of disease are often recommended to improve diagnostic accuracy.

In this particular case, the evolution of the erythroderma in the patient appeared to follow that of the underlying cancer. It has been reported that skin rashes can provide the first clue to a diagnosis in 1% of internal malignancies [12]. The cutaneous signs continued while the cancer remained resistant to therapy. This association emphasizes the idea that effective control of paraneoplastic EE is dependent on successful treatment of the underlying tumor. In situations of chronic or unexplained erythroderma, physicians should keep a high suspicion for cancer and explore less frequent primary sites, such as the central nervous system, especially if new neurologic symptoms occur. Similar presentations have been described in other malignancies, including rare reports of EE occurring with gallbladder adenocarcinoma, where the skin presentations appeared before the underlying cancer was found [13]. Recognizing this relationship may help clinicians interpret persistent or worsening cutaneous manifestations as a signal of underlying malignancy. 

## Conclusions

This case demonstrates that refractory EE may serve as a manifestation of an underlying malignancy, even when initial evaluation does not reveal anything. The delayed detection of the underlying malignancy emphasizes the importance of regular diagnostic evaluation in patients with chronic or unexplained EE, especially if additional systemic symptoms occur. Having a broad differential diagnosis may contribute to early detection of occult cancer and enhance overall patient treatment.
